# Wall segmentation in 2D images using convolutional neural networks

**DOI:** 10.7717/peerj-cs.1565

**Published:** 2023-09-11

**Authors:** Mihailo Bjekic, Ana Lazovic, Venkatachalam K, Nebojsa Bacanin, Miodrag Zivkovic, Goran Kvascev, Bosko Nikolic

**Affiliations:** 1 Everseen, Belgrade, Serbia; 2 Daon, Belgrade, Serbia; 3 Department of Applied Cybernetics, University of Hradec Králové, Faculty of Science, Hradec Králové, Czech Republic; 4 Department of Informatics and Computing, Singidunum University, Belgrade, Serbia; 5 University of Belgrade, School of Electrical Engineering, Belgrade, Serbia

**Keywords:** Semantic segmentation, Wall segmentation, Encoder-decoder, ADE20K, PSPNet

## Abstract

Wall segmentation is a special case of semantic segmentation, and the task is to classify each pixel into one of two classes: wall and no-wall. The segmentation model returns a mask showing where objects like windows and furniture are located, as well as walls. This article proposes the module’s structure for semantic segmentation of walls in 2D images, which can effectively address the problem of wall segmentation. The proposed model achieved higher accuracy and faster execution than other solutions. An encoder-decoder architecture of the segmentation module was used. Dilated ResNet50/101 network was used as an encoder, representing ResNet50/101 network in which dilated convolutional layers replaced the last convolutional layers. The ADE20K dataset subset containing only interior images, was used for model training, while only its subset was used for model evaluation. Three different approaches to model training were analyzed in the research. On the validation dataset, the best approach based on the proposed structure with the ResNet101 network resulted in an average accuracy at the pixel level of 92.13% and an intersection over union (IoU) of 72.58%. Moreover, all proposed approaches can be applied to recognize other objects in the image to solve specific tasks.

## Introduction

The rapid evolution of deep neural networks, accessibility of large amounts of data and leveraged computing power enabled solving very complex challenges that fall into the domain of computer vision. One particular challenge in this field is image segmentation (Minaee et al., 2021), which performs the classification of every pixel of an image into a set of predefined categories. This process is also regarded as a pixel-level classifying task. Opposite to the classifying process, where models identify what is in the image, image segmentation models also conduct localization. Image segmentation comes in two main shapes: semantic segmentation and instance segmentation (Gu, Bai & Kong, 2022). The properties of the image segmentation process make it extremely versatile and applicable in a wide range of domains, that include autonomous driving (Tran & Le, 2019), agriculture (Singh, Rawat & Ashu, 2021), robotic navigation (Koval, Zahorodnia & Adamiv, 2019), medical imaging and computer-aided diagnostics (Siddique et al., 2021), satellite imagery (Neupane, Horanont & Aryal, 2021), scene understanding (Barchid, Mennesson & Djéraba, 2021), background segmentation (Karbowiak & Bobulski, 2022), *etc*. This article focuses on the task of indoor scene parsing. Moreover, it signifies the sustained development of the research presented in the conference article (Bjekic & Lazovic, 2022).

The main role of scene parsing is segmenting the given image into regions related to the semantic categories (Zhou et al., 2017, 2019). During class prediction, as well as the estimation of the location and the object’s shape within the image, an outright understanding of the observed scene is also provided.

Wall segmentation is a special case of semantic segmentation. The task is to classify each pixel into one of two classes: wall and no-wall. The goal is to distinguish walls from furniture, decor, ceilings, windows, paintings, doors, floors. The segmentation model returns a mask showing where objects like windows and furniture are located, as well as walls. This allows the system to distinguish walls from different objects and erase the items from textured wall planes. Wall segmentation is a challenging task. Owing to their resemblance to other semantic components of the indoor environment, the wall borders are typically difficult to discern. Also, there are often blurred parts of an image, representing items hanging on the wall that are difficult to localize, which makes it difficult to segment walls. These segments can be, for example, decorations on the walls (such as a clock, a photo frame and a switch), or the potted vegetation leaves in front of the walls.

The research aims to develop a system capable of segmenting walls in images of indoor scenes. The semantic segmentation of indoor areas is regarded as a very complex challenge, concerning the high data variability caused by cluttering, often with a significant variation in lighting (Liu et al., 2019). An additional problem is the resemblance of the walls to the ceilings and other similar semantic pieces, which significantly complicates the classification task. In the literature, one can find different approaches and solutions for semantic segmentation, such as the solution proposed by Zhao et al. (2017). On the other hand, architectures based on transformers are the most popular today, as Liu et al. (2022), Chen et al. (2022), Wei et al. (2022), Bao, Dong & Wei (2021) and BEIT model (Wang et al., 2022). All the mentioned systems during the evaluation use the ADE20K dataset, and the results obtained are the mIoU from 41.68% to 62.8%. Of the systems that deal with the problem of wall segmentation, the best results were achieved by magic wall research (Liu et al., 2019) and Wallnet (Huang et al., 2020) with mIoU from 68.65% to 70.41%. The main contributions of this work for wall segmentation are:

- Proposed structure based on an encoder-decoder architecture with Res-Net101 as an encoder,
- Analysis of three different approaches for model training,
- Validation of the effectiveness of the proposed model on the public ADE20K dataset. The authors obtained the best performance compared to existing approaches: accuracy at the pixel level of 92.13% and IoU of 72.58%.

The results demonstrate that the proposed model can effectively address the problem of wall segmentation.

The remainder of this article is structured as follows: an overview of the relevant work is discussed in the ‘Related work’ section, with more semantic and wall segmentation details. The ‘Proposed wall segmentation method’ section overviews the dataset, model, and proposed wall segmentation solution. The empirical results are shown in the ‘Experiment’ section. The ‘Discussion’ section discusses the proposed solution, its advantages and weaknesses, and a comparison to other published articles and work. Conclusions are given in the ‘Conclusion’ section.

## Related work

The semantic segmentation task involves designating a class label for each pixel within the image. This process handles multiple objects with the same label as a single entity. On the other hand, instance segmentation handles objects in to the same class as separate instances.

### Architectures

**Figure 1:**
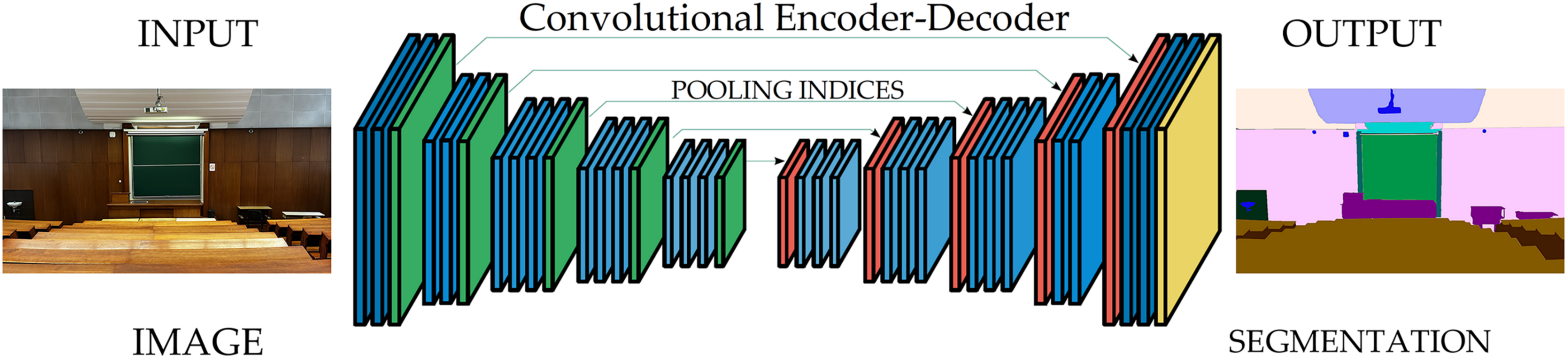
A typical architecture for semantic segmentation: Conv+BatchNorm+ReLU (Blue), Pooling (green), Upsampling (red), Softmax (yellow). Photo credit: Goran Kvascev.

The most popular architectural approach to performing semantic segmentation is symmetric. This type of architecture comprises an encoder and a decoder, coupled with a pixel-wise classifier, as shown in Fig. 1.

The traditional semantic segmentation approaches that utilize this architectural style are SegNet (Badrinarayanan, Kendall & Cipolla, 2017), U-Net (Ronneberger, Fischer & Brox, 2015; Nguyen et al., 2021), DeepLab (Chen et al., 2017), *etc*. The encoder element in this architecture consists of the pre-trained classification network that extracts the compound semantic features. However, as preserving the original image dimensions across the complete network is regarded as very intensive from the computing point of view, the encoder executes the downsampling procedure of the original input image resolution. The encoder operation results in a low-resolution feature map adjusted for efficient class discrimination. Inevitably, a significant portion of the information in the input image needs to be recovered due to the downsampling.

The role of the decoder network is to recreate the image details from the received feature map. The decoder takes the encoder output as input with the optional utilization of the additional feature maps from the encoder’s middle layers using skip connections. This procedure will aid the decoder in preventing the loss of information introduced by the encoder element. Finally, the decoder performs up-sampling the encoded attributes to the original image resolution and producing the segmentation mask as the output.

### Previous solutions

The best known metrics for evaluating semantic segmentation models are pixel accuracy (PA) and intersection over union (IoU).

The ratio of properly classified pixels to the overall number of pixels in the image is known as pixel accuracy. In the case of multiple classes, mean pixel accuracy (mPA), which describes the class average accuracy, is used. Using this metric in the case of unbalanced class datasets is not recommended because only correctly classifying the dominating class will produce high accuracy.

Intersection over union calculates the ratio between the overlap between the ground truth and the output segmentation mask, and their union. In the case of multiclass datasets, mean intersection over union (mIoU) is used. mIoU is calculated by averaging the IoU over all classes. The capability of global context information by different-region-based context aggregation was exploited (Zhao et al., 2017). An effective pyramid scene parsing network for complex scene understanding is proposed. They expanded the pixel-level feature to include the particularly adapted global pyramid pooling layer alongside the conventional dilated FCN for pixel classification. A single PSPNet yields an accuracy of 85.4% on the dataset PASCAL VOC 2012.

In recent years, models based on transformers have shown the best results. This deep learning model adopts the differentially weighing the significance of each part of the input data. Liu et al. (2022) introduced methods for scaling the Swin Transformer up to 3 billion parameters and enabling it to train with images of high resolution, reaching even 1,536 × 1,536 pixels. These strategies include the respost-norm and scaled cosine attention focused on rendering the model more conveniently expanded in capacity, and a log-spaced continuous relative position bias attitude to enable the model to be transferred more effectively throughout window resolutions. The improved model is referred to as Swin Transformer V2. They conducted experiments on ADE20K semantic segmentation dataset and Swin Transformer V2 yields mIoU of 59.9%. ADE20K consists of scene-centric images with 150 classes, including elements such as sky, roadway, grassland, and discrete entities including bed, person, *etc*.

Chen et al. (2022) presented an adapter for Vision transformers that bridges the performance gap on dense prediction tasks. It achieved comparable performance to vision-specific models by introducing inductive biases *via* a different architecture. Their ViT-Adapter-L generates a 60.5% mIoU on the ADE20K benchmark for semantic segmentation.

Wei et al. (2022) presented a simple feature distillation approach that can generally improve the finetuning performance of many visual pre-trained models. Finetuning allowed contrastive-based self-supervised learning techniques to be equivalent to cutting edge masked image modeling (MIM) techniques. It also improved a CLIP pretrained ViT-L model to reach 89.0% top-1 accuracy on ImageNet-1K classification. On ADE20K dataset improvement on SwinV2-G was from 59.9% to 61.4% for mIoU.

BEIT (BERT Pre-Training of Image Transformers) is a self-supervised pre-training framework for vision Transformers that provide significant fine-tuning performance on downstream tasks including image classification and semantic segmentation (Bao, Dong & Wei, 2021). The authors performed multimodal pre-training cohesively using similar goals and common architecture for texts and visuals. Even though BEIT does not necessitate annotations for pre-training, the suggested strategy outperforms supervised pretraining in terms of efficiency. With 25 K pictures and 150 semantic categories, they used the ADE20K benchmark to test BEIT, and the mIoU score was 47.7%. Later research on the BEIT model led to even better results (Wang et al., 2022)-on the ADE20K benchmark with mIoU equals 62.8%.

WallNet (Huang et al., 2020) is a method for reconstructing the full-room layout using a sparse image. This article demonstrates the algorithm’s efficiency in layout estimations (normal, semantic, geometric, *etc*.). The described PSPNet inspires the described methods ResNet50/101 as a basic feature extractor. The wall matching feature encodes the information about everything and the possible contextual cues for matching walls, including the furniture placement and relationship with other walls. The accuracy for normal+semantic estimation of walls was 86.84%.

A Magic-wall technology for autonomously altering the wall color of interior environment imagery has been proposed (Liu et al., 2019). To accomplish this aim, the authors suggested an edge-aware fully convolutional neural network and an improved network for accurately identifying the wall section. The Magic-wall can recognize wall zones automatically and seamlessly replace the existing color of the walls with the desired color. The entire wall color modification procedure, including wall fragmentation and color substitution, is guided by visual semantics. The researchers suggested an Edge-aware-FCN, in which a new edge-prior branch has been incorporated into edge prediction, to better detect the edges of the wall areas. Additionally, they included an Enhanced-Net to improve the fragmentation grade of wall sections even more. Nonetheless, the Edge-aware-FCN is the first point the imagery propagates through. Concatenating the generated semantic confidence map with the RGB picture creates a new input for the Enhanced-Net. In such a scenario, the Enhanced-Net may benefit from the earlier semantic-aware knowledge extracted by the Edge-aware-FCN and is incentivized to concentrate on refining more demanding and obscure specifics surrounding wall areas. The authors expand upon the ADE20K to provide a new dataset for the model assessment. The entire picture lacking the “wall” class was initially eliminated. The researchers then chose four semantic descriptors commonly alongside the walls, including floor, ceiling, window, and table. Ultimately, 3,000 photos are obtained, of which 2,500 and 500 are utilized for training and testing, correspondingly. A mIoU of 70.41% is generated by their network.

## Proposed wall segmentation method

This section describes a semantic segmentation model with a pyramid pooling module (PPM) (Zhao et al., 2017). The main blocks of the proposed structure are the model, encoder and decoder. Portions of this text were previously published as part of a conference article (Bjekic & Lazovic, 2022).

### Model

**Figure 2:**
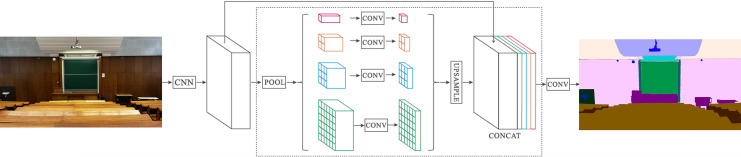
Overview of the semantic segmentation model with pyramid pooling module (PPM). Photo credit: Goran Kvascev.

This study employs a PSPNet-based encoder-decoder semantic segmentation model. PSPNet is an effective scene parsing network for complex scene understanding, which consists of several parts: CNN as an encoder, a pyramid parsing module followed by a convolution layer, as shown in Fig. 2. The model is designed to accept arbitrary sizes of input images and to output the segmentation mask of the same size. Individual layers’ input/output sizes depend on the input image size and are re-downscaled feature representations of the image.

The encoder produces a feature map from the image. Subsequently, a pyramid parsing module is employed to capture distinct sub-region representations. This is followed by upsampling and concatenation layers, which combine to create the ultimate feature representation. This representation encompasses both local and global context information. The final convolution layer generates the final per-pixel classification (Zhao et al., 2017). A trained ResNet (He et al., 2016) model with dilated convolutions (Chen et al., 2017; Yu & Koltun, 2015) is used to extract the feature map. In short, using the input image, the encoder generates a low resolution, grained feature map, and the decoder upsamples it to develop a full-resolution segmentation mask.

**Encoder:** It is common practice to deploy a customized convolutional neural network as an encoder while performing classification tasks. The ResNet50 and ResNet101 networks are employed in this study. Numerous methods for enhancing the functionality of the current semantic segmentation architectures are suggested (Chen et al., 2017). These methods enable better results to be achieved with less computational effort. One of the enhancements is applying a dilated convolution within the encoder network rather than a conventional convolution.

Fewer model parameters are required when dealing with low-resolution feature maps. A broad receptive field that makes it possible to retrieve more contextual details is another benefit. However, the fundamental drawback of low-resolution feature maps is the need for more spatial information, which is crucial for acquiring precise details for semantic segmentation.

**Figure 3:**
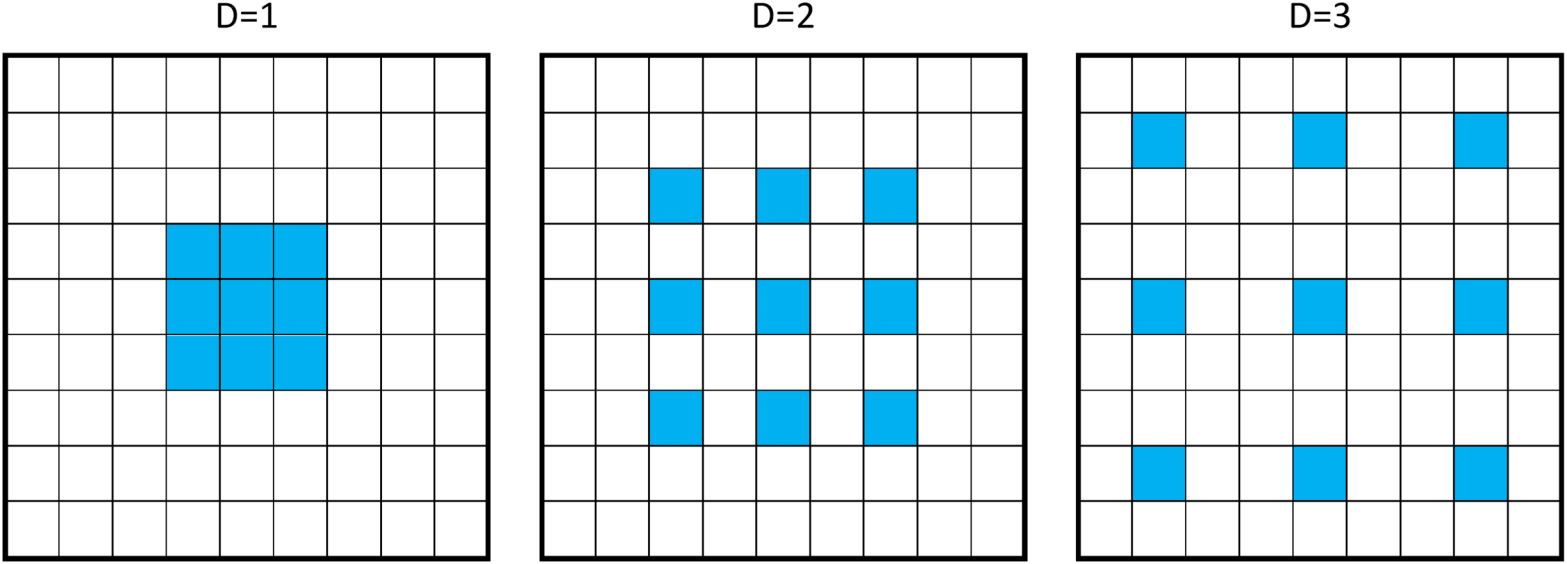
Example of 3 × 3 dilated convolution with dilation rate D = 1, 2, 3.

While maintaining spatial resolution, dilated convolution makes achieving a wide receptive field possible without introducing extra parameters. Figure 3 illustrates the dilated convolution with a $3 \times 3$ kernel size and various dilation rates.

In this article, dilated ResNet50 and ResNet101 networks are used. Following the work of Chen et al. (2017), several improvements were introduced and implemented, such as

- In the last two building blocks of the network, the stride is reduced to 1.
- All the following convolutions are replaced with dilated convolutions with a dilation rate of $D = 2$.

These changes led to better performance in the wall segmentation task. Following a comprehensive assessment of the available image database, modifications were suggested. These proposed modifications were created after analyzing the results obtained by performing many experiments on the existing database of images. Also, three different approaches and two different dimensions of the network (ResNet50/101) were applied to the research. They analyzed the results obtained with all the mentioned approaches, leading to the chosen solution (Third approach-ResNet101).

By applying them, the authors obtained the best performance (PA and IoU) for the proposed model for the wall segmentation problem.

**Figure 4:**
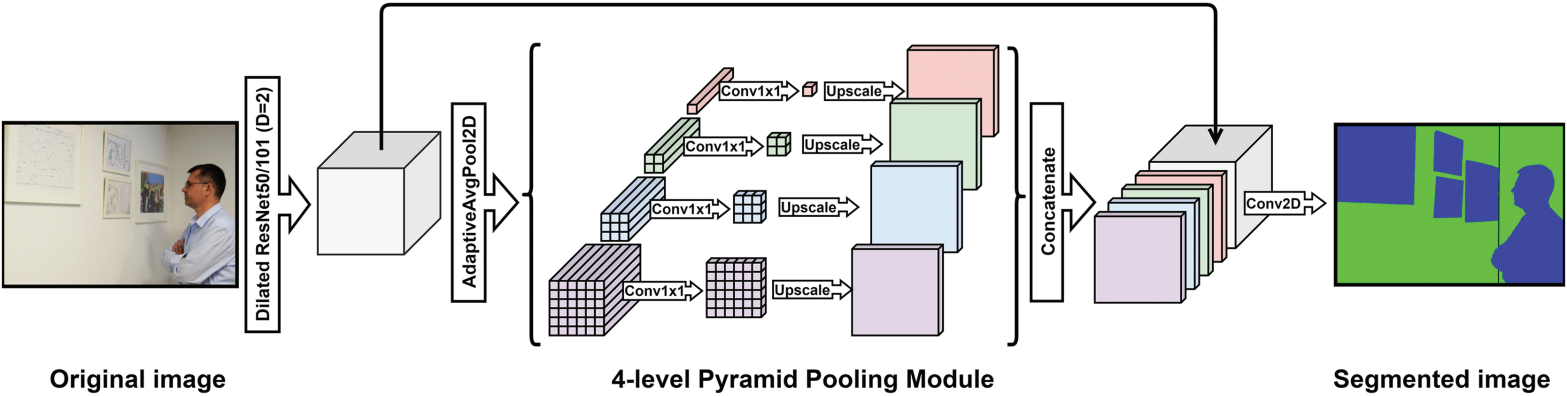
Proposed structure of the used semantic segmentation model, with the Pyramid Pooling Module. Photo credit: Goran Kvascev.

**Decoder:** The main part of the decoder is the pyramid pooling module (PPM). The entire structure of the used semantic segmentation model, with the PPM, is shown in Fig. 4.

PPM uses several region-based context aggregations to collect global context information. Adaptive pooling and 4-level pyramid pooling are layered on top of the encoded feature map. The outputs of the various pyramid module levels are feature maps of various sizes. Average pooling is applied on top of these feature maps, followed by 1 × 1 convolution. Using this convolution, where N is the number of pyramid levels, the number of channels will be decreased by an amount equal to N times compared to the feature map generated by the encoder. Bilinear interpolation is used for upsampling the extracted feature maps to the size of the input feature map. To create a global feature map, the input feature map and the four remaining feature maps are ultimately synthesized convolutional layer is applied to make the final prediction map.

Depending on the size of the feature map which is an input to PPM, the number of pyramid levels and the size of each level may be altered. The pooling filters cover the full picture, half of the picture, and quarters using a 4-level pyramid. Due to this, data collected by the PPM is more accurate than data collected *via* global average pooling. The segmentation mask is upsampled to the resolution of the input image using bilinear interpolation.

## Experiments

The dataset and experimental results of the suggested solution strategy are stated in this section.

### Dataset

The ADE20K dataset is modified and utilized in research (Zhou et al., 2017). More than 20,000 photos of interior and exterior situations, labeled with 150 distinct categories, comprise the original ADE20K dataset. Each image has an associated segmentation mask. Additionally, the components of the majority of items are indicated.

The ADE20K dataset is adjusted only to include interior images since it incorporates useless images for wall segmentation. Images of interest make up just a third of the given data. Just three labels—wall, no-wall, and unlabeled pixels are retained. The specified semantic segmentation model’s first tentative findings were implemented in PyTorch (Bjekic, 2022) and demonstrated at a conference (Bjekic & Lazovic, 2022).

Cross-entropy averaged over all spatial points in the feature map served as the criteria function for the model training process. During training, pixels that were not labeled were disregarded. The model training was conducted using three different methods.

For the classification problem, a cross-entropy loss is the preferred loss function. Pixel-wise cross-entropy loss is a commonly employed loss function because semantic segmentation involves pixel level classification (Jadon, 2020). Dice loss is a common loss function that effectively addresses the issue of imbalanced data in semantic segmentation. Yet, this loss overlooks the disparity between “easy” and “difficult” samples and solely tackles the foreground-background discrepancy. It is predicated on the dice coefficient, a metric for mask crossover.

### Training

Stochastic gradient descent (SGD) is an optimization technique used for training. The “Poly” learning rate approach was applied:

(1) $${\alpha _{curr}} = {\alpha _{start}}{\left( {1 - {{iter} \over {\;ma{x_{iter}}}}} \right)^{0.9}}$$

The maximum number of iterations was chosen as $ma{x_{iter}} = 100,\!000$ and the beginning learning rate was fixed at $\alpha = 0.02$. The $iter$ parameter returns the current iteration. There were 20 epochs with 5,000 iterations in each epoch.

Stochastic mirror flip and arbitrary resizing into one of the predefined sizes were used for data augmentation. Before the last convolutional layer in the decoder section, dropout with the value $p = 0.1$ was carried out as an extra regularization. Additionally, each batch contains two pictures.

The research was conducted in two phases. In the first phase, three model training methods using the dilated ResNet50 network as the encoder, due to the efficiency of training and the speed of network execution, were tested. After that, the best method was chosen and the results were evaluated using the more complex ResNet101 architecture. The first model training method consists of two distinct phases. Before performing transfer learning on the modified ADE20K dataset, the model was trained on the complete ADE20K dataset (including all 150 classes). In the initial training, the decoder was arbitrarily initialized *via* Kaiming initialization, while the encoder was initialized with weights from the ResNet50 model that had been pre-trained on ImageNet. To perform transfer learning, the final output layer of the decoder was modified (to permit classification into two classes instead of 150 classes), and only this new layer was trained while the previous layers were frozen. Following the transfer of the weights, the model was trained for just a single epoch.

In contrast to the previous strategy to model training, after training the model on the entire ADE20K dataset, the second approach trained the decoder as the whole structure, not just the last layer, while the encoder weights were fixed. The modified model was then trained for five epochs. The third method commenced with the modified ADE20K dataset. There was no transfer learning, in contrast to the earlier strategies. The model was trained end-to-end having two classes after instantiating the encoder with pre-trained ResNet50 and stochastic initialization of the decoder. Since the third method with the ResNet50 network achieved the best preliminary results among these three approaches, additional training using the ResNet101 network as the encoder and the accompanying analysis were performed. This ResNet101 network, called “end2end” learning, was trained without transfer learning.

### Results

The subset of the modified ADE20K dataset, which exclusively includes indoor images, is used for model validation. Pixel accuracy (PA) and intersection over union (IoU) are the metrics employed for the model performance analysis.

Table 1 provides metrics of models trained using the three alternative approaches and two types of ResNet architectures.

**Table 1:** Evaluation results on the validation set.

	First approach	Second approach	Third approach	Third approach
	ResNet50	ResNet50	ResNet50	ResNet101
PA (%)	84.82	86.24	90.75	**92.13**
IoU (%)	56.87	59.08	69.05	**72.58**
Execution time (ms)	11.4	11.4	11.4	18.8

**Note:**

The bold values indicate the best method/score.

From the findings in Table 1, it is evident that the third technique to model training, in which the model was customized to identify solely two classes from the beginning, yields the best pixel accuracy and IoU. Remembering high pixel accuracy doesn’t always imply that the segmentation model performs efficiently for each class, particularly in datasets with unbalanced classes. Because of this, IoU is the preferred metric.

**Figure 5:**
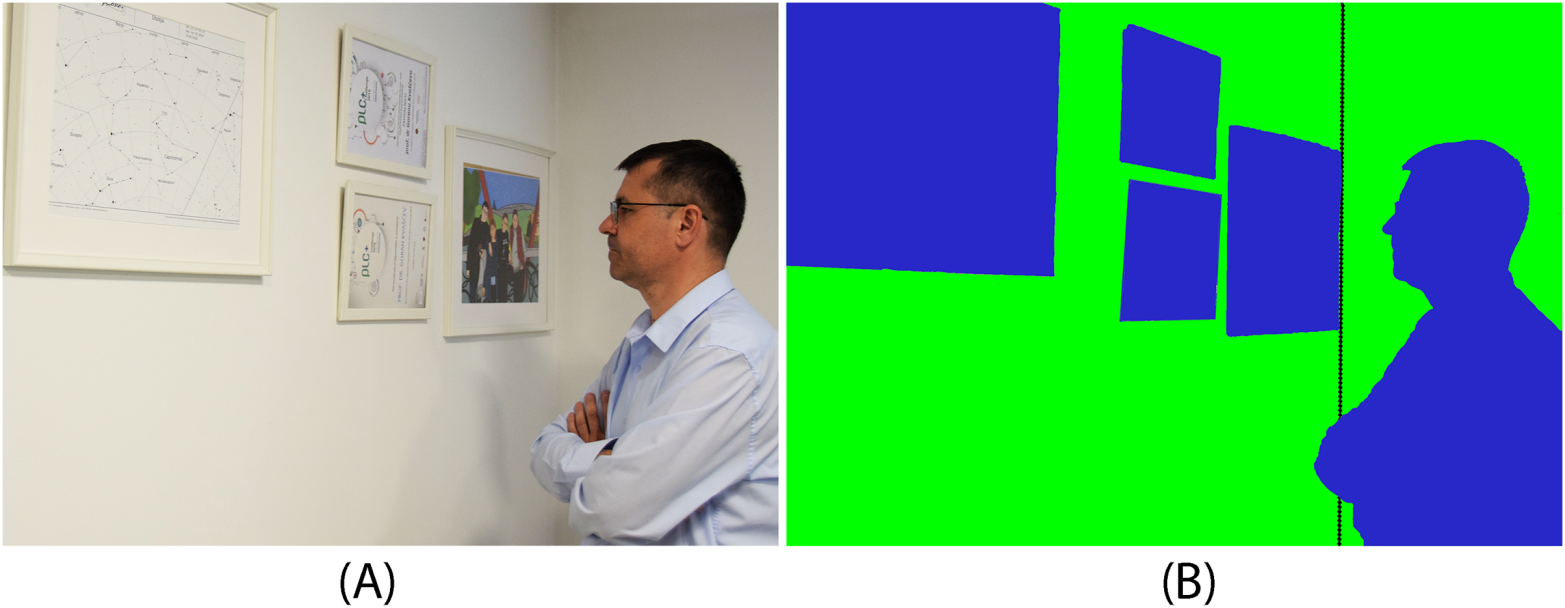
Sample of one image from a dataset with associated segmentation masks (green–wall, blue–no-wall). Original image (A) and ground truth (B). Photo credit: Goran Kvascev.

**Figure 6:**
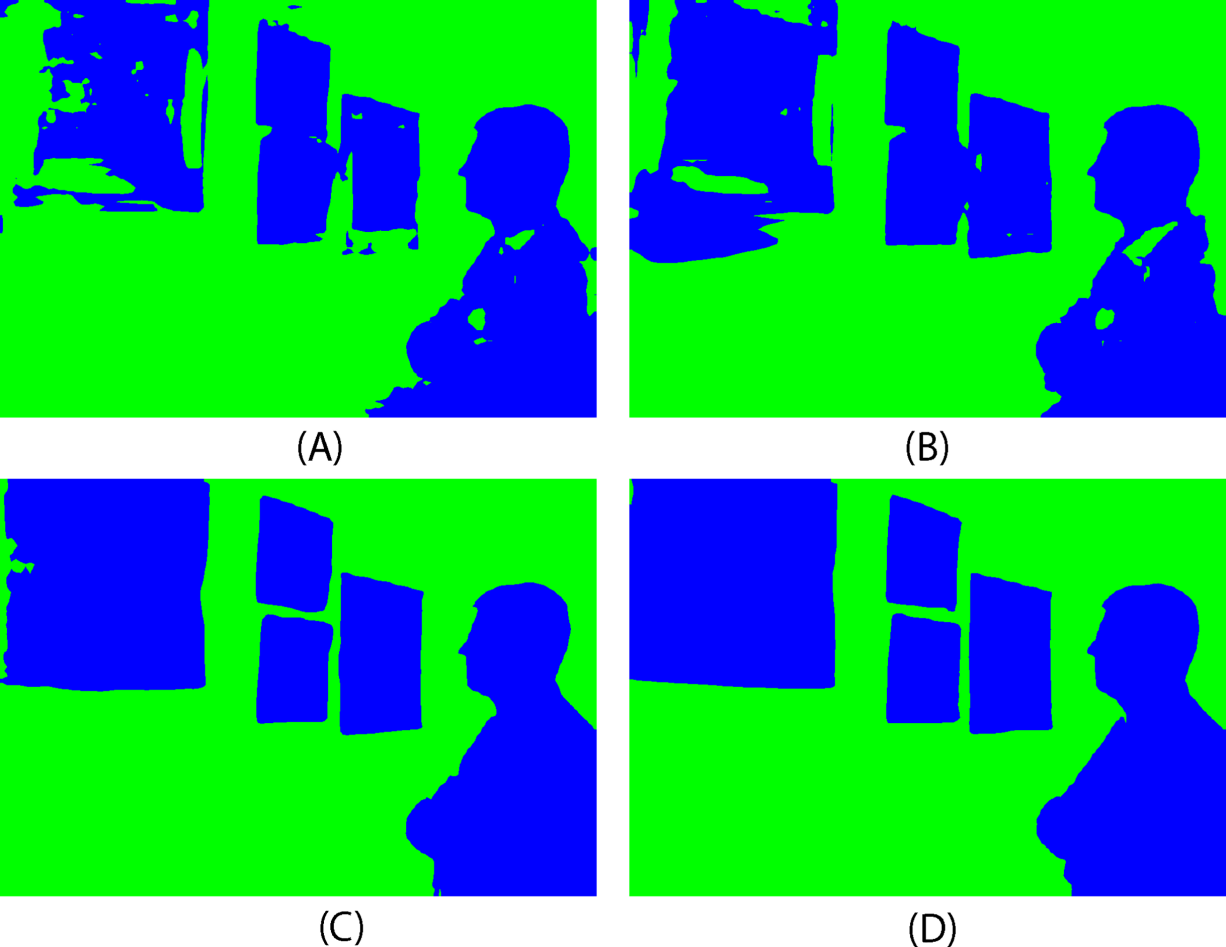
Predicted segmentation masks: (A) first approach-ResNet50, (B) second approach-ResNet50, (C) third approach-ResNet50, (D) third approach-ResNet101, (green–wall, blue–no-wall).

For one new image, made by authors (Bjekic, 2022), the outcomes of wall segmentation for each of the three strategies are shown below, along with the associated pixel accuracy and intersection over union values. The original image and ground truth are presented in Fig. 5 while the predicted segmentation masks are displayed in Fig. 6, for three approaches and ResNet50 and ResNet101 as encoders.

The average execution times for processing a single image using a CPU are shown in Table 1. Since the first, second and third approaches with ResNet50 have an identical structure, it is reasonable to expect that their execution times would be practically the same. As the third approach ResNet101, has almost twice the number of parameters compared to the other approaches, it is reasonable to expect that its execution time would be almost twice as long.

Based on the previous images, it can be seen that the first approach gives the worst results. Many pixels of paintings are classified as walls. There is an improvement using the second approach, but the third approach provides the best results.

**Figure 7:**
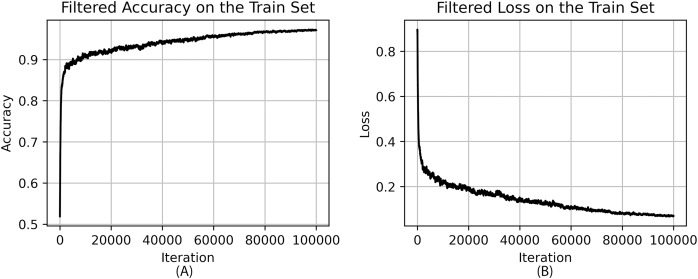
Accuracy (A) and loss (B) of the best model on the train set during training.

Figure 7 shows filtered accuracy and filtered loss of the best model on the train set during training at each iteration.

**Figure 8:**
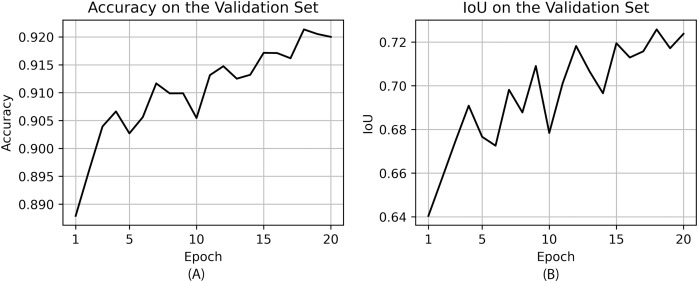
Accuracy (A) and IoU (B) on the validation set during training.

In Fig. 8, pixel accuracy and IoU on the validation set for each epoch during training are shown.

The study used a specialized computer system designed for training deep networks and implementing the proposed structure. The system had the following specifications: an Intel i9-12,900 processor-based PC, 32 GB DDR4 memory, and a GeForce RTX 3080 Ti with 12 GB GDDR6, capable of executing algorithms.

## Discussion

During model testing, it has been observed that different limitations are imposed mostly by data quality. Some limitations are discussed in more detail below.

All data in the ADE20K dataset are grouped into different scene categories, such as living room, bedroom, church, airport, *etc*. A subset of scene categories was selected when creating a modified ADE20K dataset used for training, described in this article. This selection was made assuming that images of a certain category contain walls. There was no validation of whether the selected images included walls or not. As a result, some images in the final dataset could not be interesting for training the wall segmentation model. This may result in model performance degradation.

During error analysis, it has been noticed that certain images have either wrongly annotated walls or pixels of wall regions not annotated at all. Another dataset related problem that may affect the model quality is when a scene in the image is cluttered with various objects.

The model is trained on various resolutions and gives the best results for images of similar resolutions. The model does not behave as expected for images of substantially different resolutions. When the input resolution is large, the image should be downsampled to a lower resolution within the range the model was trained on. On the other hand, if the input resolution is too small, the model cannot extract all the information necessary for segmentation from the image.

Regarding semantic segmentation, human error performance is a good proxy for the Bayes error (Mason, 2022). So, if humans cannot successfully distinguish between wall and no-wall classes in an image, it cannot be expected from the model to perform well on this image.

The next point is the accuracy analysis of the proposed algorithm. The best-known metrics for evaluating semantic segmentation models are pixel accuracy (PA) and intersection over union (IoU). Table 2 describes the results of the semantic segmentation performance of different methods applied to the ADE20K image dataset, expressed through PA and IoU indicators. However, the authors of certain previously suggested methods (Table 2, methods 4, 6, 7, 8, 9 and 10) didn’t publish their results as pixel accuracy (PA). Thus these metric values are absent from Table 2. Namely, the IoU metric is often used in the literature as a more robust indicator of unbalanced classes.

**Table 2:** Evaluation results on the validation set for different methods.

Method	Method	PA (%)	IoU/mIoU (%)	ADE20K
1	ResNet50+DA+AL+PSP (Zhao et al., 2017)	80.04	41.68	Original
2	ResNet269+DA+AL+PSP (Zhao et al., 2017)	80.88	43.81	Original
3	SwinV2-G (Liu et al., 2022)	63.1	59.9	Original
4	ViT-Adapter-L (Chen et al., 2022)	–	60.5	Original
5	FD-SwinV2-G (Wei et al., 2022)	64.2	61.4	Original
6	BEIT-L+ (Bao, Dong & Wei, 2021)	–	58.4	Original
7	BEIT-3 (Wang et al., 2022)	–	62.8	Original
8	Deeplab-ResNet101 (Liu et al., 2019)	–	68.65	Modified
9	Edge-aware-FCN-VGG16 (Huang et al., 2020)	–	65.80	Modified
10	Edge-aware-FCN-ResNet101 (Huang et al., 2020)	–	70.41	Modified
11	First approach-ResNet50	84.82	56.87	Modified
12	Second approach-ResNet50	86.24	59.08	Modified
13	Third approach-ResNet50	90.75	69.05	Modified
14	**Third approach-ResNet101**	**92.13**	**72.58**	Modified

**Note:**

The bold values indicate the best method/score.

For analyzed methods: ResNet50+DA+AL+PSP, ResNet269+DA+AL+PSP, SwinV2-G, ViT-Adapter-L, FD-SwinV2-G, BEIT-L+, BEIT-3 (numbered in the table from 1 to 7) the indicators of mIoU are shown. As these methods deal with the semantic segmentation of multiple classes within the ADE20K dataset, these results are expected to be worse than those for the wall segmentation problems. The methods entitled Edge-aware-FCN-VGG16, Deeplab-ResNet101, Edge-aware-FCN-ResNet101, First approach-ResNet50, Second approach-ResNet50, Third approach-ResNet50, Third approach-ResNet101 (numbered in Table 2, methods 8 to 14) deal with the problem of wall segmentation. For these studies, a subset of the ADE20K dataset was created. This subset consists of 3,000 images that contain walls, 2,500 and 500 images for training and testing, respectively.

The best results were achieved by the Edge-aware-FCN-ResNet101 network with IoU = 70.41%. In this article, the proposed Third approach-ResNet50 network scored IoU = 69.05% and the same approach with ResNet101 got a better result, IoU = 72.58%. The Edge-aware-FCN-ResNet101 network has a better IoU than the proposed third approach-ResNet50 by only 1.36%. Still, much better results from the significantly more complex applied neural network ResNet101 compared to the proposed structure in which the ResNet50 network is implemented. Using the proposed simpler structure ResNet50 requires half the number of floating point operations 3.8 GFLOPs *vs* 7.6 GFLOPs for ResNet101 (He et al., 2016). On the other hand the article (Zhao et al., 2017) showed that by using the more complex deep network, performance improvement in IoU could be more than 2%. The third approach with ResNet101 achieved better results with IoU = 72.58%, while sacrificing the required execution time.

A significant difference in obtained results can be observed when comparing the three approaches presented in this article. Due to the few images present during training, the initial expectation may be that transfer learning should provide the best results, which is different here. The potential reason for this can be seen when the semantic segmentation task is regarded as the classification task on each pixel. In this case, the number of examples used for training the classification model is much larger than the number of images in the dataset. Due to this observation, semantics segmentation with a few images can be observed as a classification task with many large examples. Here are enough examples for training, there is no need for transfer learning to obtain better results. The set of images used contains scenes with different light conditions. The results obtained in Table 2 also include these scenes, so the proposed method was verified in different light conditions. This constatation is by results obtained using the three approaches.

Another potential reason for worse results when using transfer learning may be that the initial training was done on 150 classes, of which many are not of interest for the task of wall segmentation. When training on 150 classes, the model also learns features that are not important for discerning between classes wall and no-wall. Potentially better results may be obtained if the initial training was done only a few classes are often associated with the wall, such as the floor, ceiling, painting, door and window.

## Conclusion

This article described a model structure for the semantic segmentation of walls. The encoder-decoder architecture was used. As the encoder, dilated ResNet50 and dilated ResNet101 networks were used. The building block of the decoder was the pyramid pooling module in combination with the bilinear interpolation. The model was trained on a modified ADE20K dataset, consisting only of interior scene images with two classes (wall and no-wall). Three different approaches to model training were tested. The best approach was directly training the model on the modified ADE20K dataset, without transfer learning. Implementation of all approaches is provided in Bjekic (2022).

Wall segmentation is a complex task, due to strong occlusions, similarity with other semantic parts of the interior scenes, and different objects that occlude the wall and are hard to localize. During model development, various problems with the current setup of the project were observed. In future work, most of these problems can be overcome. When it comes to the selected images, validation of each image, whether it is an image of interest and contains walls, should be performed. Also, all images with bad mask annotations should be discarded. Regarding images with any ambiguities, these images should be treated carefully. All ambiguous images reflect the model performance, and their influence cannot be predicted. Each image should be separately reviewed, whether to discard or keep.

Besides data cleaning, future work may also involve experimenting with different model architectures to increase validation metrics. Also, lighter models can be implemented to speed up the entire wall segmentation system and result in realtime (Liu et al., 2023). The practical application of such a system can also be explored in future work. Also, future work will be aimed in several directions: at different network architectures, processing remote sensing images (Shun et al., 2022), and 3D wall segmentation from single panoramic images (Xu et al., 2022; Rezaei, Houshmand & Fatahi Valilai, 2021).

## Additional Information and Declarations

### Data Availability

The following information was supplied regarding data availability: The ADE20K dataset is available at MIT CSAIL: https://groups.csail.mit.edu/vision/datasets/ADE20K/. The code is available at GitHub and Zenodo: - https://github.com/bjekic/WallSegmentation. - Mihailo Bjekić, Ana Lazović, & Emilio Rodriguez. (2023). bjekic/WallSegmentation: Initial release (v0.1.0). Zenodo. https://doi.org/10.5281/zenodo.7944202.
